# Epigenetic modulation of visceral nociception

**DOI:** 10.1111/nmo.14443

**Published:** 2022-08-10

**Authors:** QiQi Zhou, George Nicholas Verne

**Affiliations:** ^1^ Department of Medicine University of Tennessee College of Medicine Memphis Tennessee USA; ^2^ Memphis VA Medical Center Research Service Memphis Tennessee USA

**Keywords:** abdominal pain, amygdala, disorders of brain‐gut interaction, DNA methylation, epigenetics, irritable bowel syndrome, visceral hypersensitivity, visceral pain

## Abstract

Epigenetics is a process that alters gene activity or phenotype without any changes in the underlying DNA sequence or genotype. These biological changes may have deleterious effects and can lead to various human diseases. Ongoing research is continuing to illuminate the role of epigenetics in a variety of pathophysiologic processes. Several categories of epigenetic mechanisms have been studied including chromatin remodeling, DNA methylation, histone modification, and non‐coding RNA mechanisms. These epigenetic changes can have a long‐term effect on gene expression without any underlying changes in the DNA sequences. The underlying pathophysiology of disorders of brain‐gut interaction and stress‐induced visceral pain are not fully understood and the role of epigenetic mechanisms in these disorders are starting to be better understood. Current work is underway to determine how epigenetics plays a role in the neurobiology of patients with chronic visceral pain and heightened visceral nociception. More recently, both animal models and human studies have shown how epigenetic regulation modulates stress‐induced visceral pain. While much more work is needed to fully delineate the mechanistic role of epigenetics in the neurobiology of chronic visceral nociception, the current study by Louwies et al., in *Neurogastroenterology and Motility* provides additional evidence supporting the involvement of epigenetic alterations in the central nucleus of the amygdala in stress‐induced visceral hypersensitivity in rodents.

## 1 INTRODUCTION

Chronic abdominal pain is one of the most common gastrointestinal symptoms in the United States and leads to significant morbidity. Our knowledge of the underlying pathophysiology of disorders of gut‐brain interaction (DGBI) such as irritable bowel syndrome (IBS) is still largely unknown.^1^, ^2^, ^3^, ^4^ Chronic abdominal pain occurs in over 100 million people each year in the United States at a cost of over $635 billion annually.^5^ Available treatments for chronic abdominal pain are far from ideal as the pathophysiological mechanisms of visceral pain are still poorly understood.^6^, ^7^ Noxious environmental stimuli, tissue damage, enteric infections, and inflammation can all evoke enteric neuroplasticity and lead to chronic GI symptoms including abdominal pain, diarrhea or constipation, bloating and urgency.^8^, ^9^, ^10^ Transient bowel inflammation disrupts gut motility and increases visceral nociception, which persist long after resolution of the inflammation as has been demonstrated in animal models of colitis, intestinal hyperpermeability, inflammatory bowel disease, and bile acid diarrhea.^11^, ^12^, ^13^ More recently, epigenetic changes have been shown to play an important role in chronic visceral nociception following noxious insults to the gut. The following paper will review evidence for DNA methylation as an epigenetic mechanism leading to stress‐induced neuroplasticity and chronic visceral nociception in patients with DGBI and in animal models of stress‐induced visceral hypersensitivity.

## 2 THE NEUROBIOLOGY OF STRESS

Stress is a well know adaptive response threats to the body. Many factors modulate an individual's stress response including previous experiences to stressors, gender, ethnicity, hormonal state, and age. The stress response is very complex and includes multiple overlapping and redundant mediators including neuropeptides, steroid hormones, and neurotransmitters.^14^ The adaptive response to stress also involves modulation of neuronal functioning at multiple levels in the brain which involve memory, decision making, emotional, autonomic, and hormonal responses.^15^ The stress response is part of the innate response that allows fine tuning of the brain's adaptation to both acute stress‐provoked challenges that are critical for short term survival, to storage of memory for long‐term responses if similar events are repeated in the future.^14^

Stress results in an alteration in both synaptic plasticity in the brain and neurotransmission through the hypothalamic–pituitary–adrenal axis (HPA). The HPA axis coordinates both short and long‐term responses to stress through several central mediators including glucorticosteroids and corticotropin‐releasing hormone that modulate behavioral, cellular, and physiological changes and adaptations.^14^ The neuroendocrine response to high stress conditions will often be marked by increased levels of cortisol as a result of increased activity of the HPA axis. It has been well established that stress plays an important role in the underlying pathophysiologic features of DGBI and IBS.^16^, ^17^ Significant increases in cortisol and adrenocorticotropic hormone have also been demonstrated in IBS patients compared to healthy individuals following nociceptive pain testing.^18^ These results suggest that IBS patients experience these pain stimuli as more stressful compared to normal individuals.^19^

## 3 ROLE OF STRESS IN DISORDERS OF BRAIN‐GUT INTERACTION

Evidence suggests that early adverse life events are tightly correlated with the initiation and severity of gastrointestinal symptoms in IBS.^20^ Early adverse life events have been shown to lead to an enhanced stress‐response of the HPA axis in IBS patients.^21^ In addition, dysregulation of the HPA axis has been shown to occur in response to hormonal challenge, psychological, or physical stress.^22^ Multiple biological systems influence pain perception, and noxious stimuli may induce changes in physiological processes, many of which serve to fine tune nociceptive afferent output. Noxious stimuli evoke a “stress response” characterized by HPA and sympathoadrenal medullary activation. A common hormonal marker of HPA responses is cortisol, which is increased following a variety of psychosocial stressors, including painful stimuli.^23^ In addition, the opioid β‐endorphin is released from the pituitary under stressful conditions.^24^

Stress‐induced pain activation of the sympathoadrenal medullary system also results in release of opioid peptides such as enkephalins from the adrenal medulla. In addition to their utility as measures of the physiological response to pain, neuroendocrine and cardiovascular responses can actively modulate pain perception. Indeed, HPA axis function influences pain sensitivity and some endogenous analgesic responses. For example, in animal models, adrenalectomy increases basal nociceptive sensitivity, and corticosterone replacement increased pain thresholds.^25^ Moreover, adrenalectomy prevented expression of opioid‐mediated foot shock‐induced analgesia, while corticosterone replacement reinstated the analgesic response.^26^ Thus, both basal pain sensitivity and endogenous analgesic responses can be influenced by HPA activation.

The endogenous opioid system in which basal opioid activity may influence pain sensitivity, is one of the most well characterized biological systems involved in the stress‐induced pain response. Higher plasma levels of β‐endorphin are associated with diminished pain sensitivity to stress while elevating β‐endorphin pharmacologically produces increased pain thresholds.^27^ In addition, endogenous opioid responses can be elicited by various environmental stimuli, including pain, and the opioid system is implicated in several intrinsic forms of analgesia, including stress‐induced analgesia, diffuse noxious inhibitory controls, and acupuncture analgesia.^28^

Stress alters neuronal activity and neuronal plasticity in several areas of the brain including prefrontal cortex, hippocampus, and the amygdala.^29^ The amygdala is a well characterized group of neurons within the limbic system and is well known to play an important role in the emotional perception of sensory stimulation.^30^ In the rat brain, the amygdala is composed of 13 nuclei, cortical regions, and corresponding subdivisions. Increased neuronal activity between brain regions including the brainstem, forebrain, and the hypothalamus occurs following nociceptive afferent input to the central nucleus of the amygdala,^31^ which has well described functional roles in emotional memory, fear, behavior, and autonomic and somatomotor responses to threatening stimuli.^32^ Increased activation of the amygdala along with the emotional arousal network has been seen in neuroimaging studies in patients with IBS^33^ with one fMRI neuroimaging study showing increased brain activation in the affective regions, including the amygdala, in response to an uncued pain expectation of a nociceptive pain stimulus.^34^ This increased activation of the amygdala and the emotional arousal network is consistent with a model of increased autonomic responses, anxiety, vigilance, that are altered in IBS patients.

## 4 EPIGENETIC MECHANISMS OF THE STRESS RESPONSE

Over the last century, we have come a long way in our understanding of epigenetic mechanisms. The term “epigenetics” was first coined by Conrad Waddington, a developmental biologist, in 1942, and over the subsequent 80 years, much work has progressed on the role of DNA methylation in disease states culminating in 2008 with the launch of the NIH Roadmap Epigenetics Program, after which we saw an explosion in knowledge related to epigenetic changes and pathophysiology.^35^ Epigenetics is a process that alters gene activity or phenotype without any changes in the underlying DNA sequence or genotype. These changes occur naturally and are influenced by age, environmental factors such as the microbiome, and disease states. Epigenetics may also manifest in how cells terminally differentiate throughout the body or can lead to diseases such as cancer, and many new studies are uncovering new roles of epigenetics in a variety of human diseases and disorders. Of specific interest to the gastrointestinal field, there are many new working models of DBGI, such as IBS, in which epigenetic changes or mechanisms are believed to play an important role in the endophenotypes of patients.

Several categories of epigenetic mechanisms that have been studied including DNA methylation, histone modification, chromatin remodeling, and non‐coding RNA. These mechanisms can have a long‐term effect on gene expression without any underlying changes in the DNA sequences. There has been a recent interest in epigenetic regulatory pathways as they play key roles in physiological pathways including regulation of the cell cycle. Since the original studies in 1969 related to its role in long‐term memory, DNA methylation has been one of the most well studied epigenetic changes, particularly over the last 2 decades, in both patients and in animal models.^35^ DNA methylation (catalyzed by DNA methyltransferases, DNMTs) plays a critical role in normal cellular function and involves the addition of a methyl group to part of the CpG island that prevents certain genes from being expressed. The key enzymes involved in DNA methylation are inducible and altered by environmental and biochemical modulation and lead to a methylation pattern that is reversible.^36^ DNA methylation, especially aberrant methylation, leads to several human disease states especially cancer‐associated pathways.^37^

## 5 DNA METHYLATION IN VISCERAL NOCICEPTION

One of the most widely studied epigenetic mechanisms in visceral nociception is DNA methylation.^38^ DNA methylation suppresses transcription factor binding at specific promoter regions through a complex comprising a DNA methylation‐dependent DNA‐binding protein (methyl‐CpG‐binding protein 2).^39^ Binding of proteins at methylated CpGs leads to increased inhibitory transcription factors which then produce downregulation or silencing of gene transcription.^40^ DNA methylation has been shown to lead to an alteration of visceral nociception in the central nervous system; increased methylation of the glucocorticoid receptor promoter in the amygdala leads to a significant decrease in the glucocorticoid receptor mRNA expression following water avoidance stress (WAS) induced visceral hypersensitivity.^41^ This experimental animal model provides evidence that chronic psychological stress induces visceral hypersensitivity through DNA methylation of genes in the central nervous system.

Recent emerging evidence suggests that DNA methylation may also play an important role in patients with DGBI such as IBS, in addition to animal models of visceral nociception. One of the first studies identified genome‐wide DNA methylation changes in peripheral blood mononuclear cells of IBS patients.^42^ DNA methylation levels were significantly higher in selected CpG (*5′‐C‐phosphate‐G‐3′*) sites of GSTM5 (glutathione‐S‐transferases mu 5), SSPO (subcommissural organ‐spondin), and TPPP (tubulin polymerization promoting protein) genes in IBS patients compared to controls. These 3 genes are involved in the serotonin system, central nervous system development, brain‐specific proteins, and glutathione and oxidative stress pathways. Further analysis revealed that epigenetic changes in the SSPO genes were positively correlated with anxiety and depression scores in IBS patients.^42^ Thus, differentially methylated gene profiles suggests that complex overlapping molecular signatures exist in IBS patients and may provide insights into important diagnostic biomarkers. More importantly, these changes may have mechanistic implications for potential epigenetic therapies for IBS patients.

Epigenetic changes have also been reported in animal models of stress‐induced visceral hypersensitivity. One such study investigated the epigenetic mechanisms that regulate chronic stress‐induced visceral pain in the peripheral nervous system of rats.^43^ In this study, lumbo‐sacral DRGs were obtained from rats subjected to 10 consecutive days of WAS. This experimentally induced stress increased corticosterone which then activated the transcription factors NR3C1:NR3C2. These were then shown to enhance transcription of DNA methyltransferases of *Cnr1* and Histone acetyltransferases of *Trpv1* that lead to decreased expression of the anti‐nociceptive endocannabinoid receptor 1 (CNR1) and increased expression of the pro‐nociceptive endovanilloid (TRPV1) respectively, which induced visceral hyperalgesia. A very interesting finding in this study is that knockdown of DNMT1 and EP300 in rat lumbo‐sacral DRGs decreased DNA methylation and histone acetylation and prevented the stress‐induced increases in visceral pain.^43^ These results pave the way to the use of gene silencing to target peripheral sensory pathways to treat abdominal pain which avoid central nervous system side‐effects.

Stress‐induced visceral hypersensitivity in female rats has been shown to be induced by down regulation of the glucocorticoid receptor in the central nucleus of the amygdala.^44^ Chronic stress alters glucocorticoid receptor expression in the central nucleus of the amygdala through DNA methylation of the glucocorticoid receptor promoter. The most recent study supporting epigenetic mechanisms by Louwies et al., in the current addition of *Neurogastroenterology and Motility* adds further support for the role of the central nucleus of the amygdala leading to stress‐induced visceral hypersensitivity in a rodent model.^45^ The current study evaluated whether chronic WAS alters DNA methylation of the glucocorticoid receptor exon 1_7_ promoter region, a region that is homologous to the human glucocorticoid receptor promoter. Administration of the HDAC inhibitor trichostatin A directly into the central nucleus of the amygdala prevented WAS induced DNA methylation of the glucocorticoid receptor exon 1_7_ promoter region. Overall, WAS increased CpG methylation of the glucocorticoid receptor promoter in the central nucleus of the amygdala in female rats which persisted for up to 28 days.^45^ Thus, alterations in DNA methylation drive regulation of central mechanisms that may lead to stress‐induced visceral hypersensitivity.

## 6 CONCLUSIONS

Substantial evidence exists for the role of epigenetic changes in stress‐induced visceral hypersensitivity in experimental animal models of nociception and in humans with chronic visceral pain. These epigenetic mechanisms may be responsible for the underlying pathophysiology of DBGIs. If future studies can further define the mechanistic role of epigenetics in the neurobiology of chronic visceral nociception, new and more targeted treatment paradigms could be developed for patients suffering from these chronic gastrointestinal symptoms. The end result would then lead to personalized medicine approaches in patients with various endophenotypes of DGBI.

## AUTHOR CONTRIBUTIONS

Both authors contributed equally to the conception, drafting, and editing of the manuscript.

## ACKNOWLEDGEMENTS

Supported by NIDDK (DK099052, DK118959) to GNV & QZ; Department of Veterans Affairs (CX001477‐01) to QZ.

## CONFLICT OF INTEREST

The authors have no conflicts of interest to disclose.
